# Quadricuspid Aortic Valve Visualized by Three-Dimensional Transthoracic Echocardiography

**DOI:** 10.1155/2011/345721

**Published:** 2011-08-10

**Authors:** Eser Acar, Tayfun Sahin, Irem Yılmaz, Umut Celikyurt

**Affiliations:** Department of Cardiology, School of Medicine, Kocaeli University, 41380 Kocaeli, Turkey

## Abstract

Quadricuspid aortic valve is a rare congenital anomaly that may cause aortic regurgitation. A 77-year-old male patient was referred to our clinic with complaints of stable angina pectoris. We report a case of a quadricuspid aortic valve diagnosed by 3-dimentional transthoracic echocardiography.

## 1. Case Presentation

**Figure 1:**
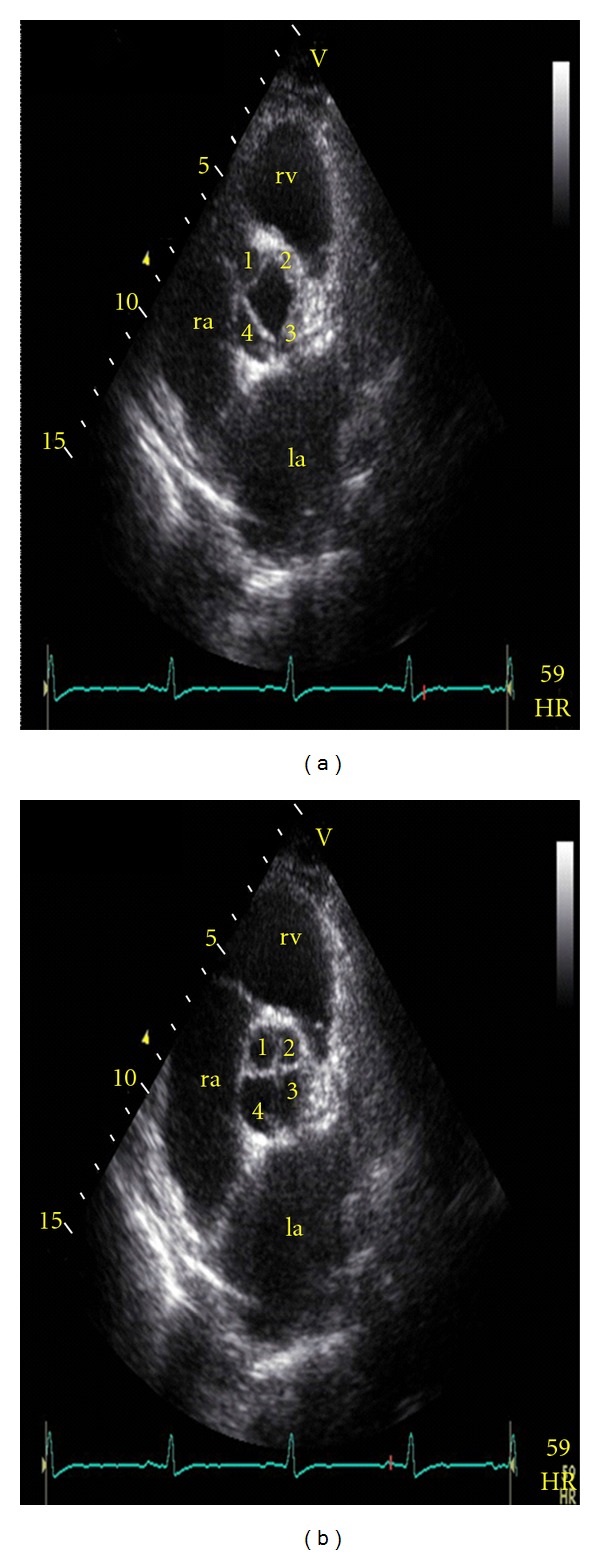
Transthoracic echocardiography images. Short axis view during systole (a) and diastole (b). ra: right atrium, la: left atrium, and rv: right ventricule.

**Figure 2:**
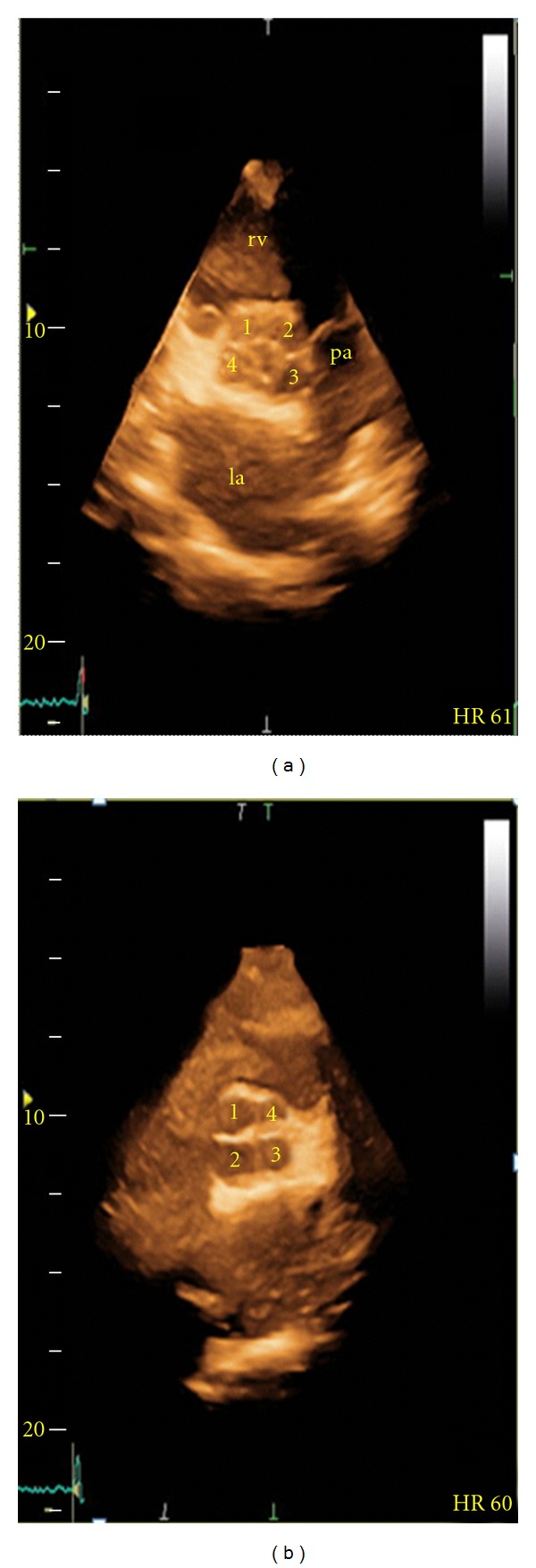
3-dimensional transthoracic echocardiography images. Short axis view during early systole (a) and diastole (b). pa: pulmonary artery, la: left atrium, and rv: right ventricule.

A 77-year-old male patient was admitted to our cardiology clinic with complaints of exertional dyspnea and angina pectoris. In the physical examination, the blood pressure was 170/100 mmHg and the pulse rate was 76 bpm. In cardiac auscultation, the heart beats were rhythmic, S1 normal, S2 hard, no S3 and S4. There was a mid diastolic murmur in the aortic focus. The other system examinations were normal. Electrocardiography showed sinus rhythm with a heart rate of 82 bpm without pathologic ST segment changes. His biochemistry tests and hemogram were in normal range. On transthoracic echocardiography (TTE) left ventricular dimension (56 mm) was at the upper limit, interventricular septum (12,9 mm) and posterior wall (12 mm) thickness increased (eccentric left ventricular hypertrophy), inferior wall segments were hypokinetic, and systolic functions were decreased (EF: 35%). On parasternal short axis view (Figures 1 and 2) the aortic valve has four cusps, the cusps were thick, its opening was enough, and its closing was irregular; thus, it causes moderate aortic regurgitation. Mitral valve cusps were thick, and there was mild mitral regurgitation. Tricuspid and pulmonary valve have no remarkable changes. On coronary angiography, in the mid-LAD 80% and in the mid-RCA 90% obstruction was detected and in the same session stents were implanted in the LAD and RCA. He was discharged with medical therapy.

## 2. Discussion

Quadricuspid aortic valve (QAV) is a rare semilunar valve malformation with an incidence of 0.008 % at autopsy and 1% in patients presented for aortic valve surgery [1]. The exact underlying mechanism of congenital QAV is not known. Aberrant fusion of the aorticopulmonary septum or abnormal mesenchymal proliferation in the common trunk may lead to abnormal cusp formation [2–4]. Although it was first detected in autopsy series in 1862, it was showed by echocardiography in 1984. Even if it is generally an isolated case, sometimes coronary arterial anomalies, ventricular septal defects (VSD), patent ductus arteriosus (PDA), and other valvular malformations can accompany it. In 1973, Hurwitz and Roberts defined seven anatomical types (types A–G) for QAV [2, 5, 6]. In our case, type A QAV, the second most common type, in which all the cusps are of equal size, is seen. We easily detected this pathology with TTE due to good image quality. However, sometimes QAV can be missed by TTE. If there is a doubt about diagnosis, real-time 3D TTE can be used for definite diagnosis. We can encompass the whole aortic root and examine at any desired level [7, 8]. Also aortic regurgitation can be assessed more reliably [7]. Fibrotic thickness due to asymmetric mechanical stress on valve and irregular fusion of the cusps results in aortic regurgitation. Aortic stenosis is very rare. The patient had moderate aortic regurgitation and so according to ESC Valvular Heart Diseases Guideline there was no indication for the aortic valve surgery [9]. Also ESC Infective Endocarditis Guideline does not suggest prophylaxis to valvular patients [10].

## 3. Conclusion

QAV is a very uncommon disease usually diagnosed during adulthood. Incidence increases with the more frequent use of TTE. Real-time 3D TTE gives more detailed information about the anatomy and the definite diagnosis.
